# Interorganizational Collaboration in Transitional Care – A Study of a Post-Discharge Programme for Elderly Patients

**DOI:** 10.5334/ijic.2226

**Published:** 2016-05-31

**Authors:** Arne Orvik, Gerd E. M. Nordhus, Susanna Bihari Axelsson, Runo Axelsson

**Affiliations:** Department of Health Sciences, NTNU, Norwegian University of Science and Technology, Ålesund, Norway; Department of Sociology and Social Work, Centre for Organization, Management & Administration, Aalborg University, Aalborg, Denmark

**Keywords:** elderly patients, integration, collaboration, interorganizational health, transitional care

## Abstract

**Introduction and aim:**

This article reports a study of a post-discharge programme for elderly patients in Norway. It took place in an intermediate ward for transitional care and was based on collaboration between a municipality and a hospital, which was part of a health enterprise. The aim of the study was to analyse the collaboration and its possible effects on the quality of patient care, and the economic efficiency of the project for the organizations involved.

**Methodology:**

A mixed-methods approach, consisting of interviews, questionnaires and analyses of official documents and statistics.

**Results:**

The collaboration was working well on the top level of the organizations, but was more problematic on the operative level. However, there were clear signs of improvement. The patients who received transitional care were more satisfied with their stay at the ward than their previous stay at the hospital. They were discharged to their homes more often and perceived to have a higher level of functioning than the hospital patients. Average costs per patient were also lower in the ward than in the hospital departments.

**Conclusion:**

The collaboration had mainly positive impacts on the quality of patient care and the economic efficiency of elderly care in the municipality. However, the board of the health enterprise decided to close down the intermediate ward.

## Background and Aims

### Interorganizational collaboration in care of the elderly

Integration has become an increasingly important issue in the development of the modern welfare society. At the same time, subspecialisation of professions in combination with decentralization has led to differentiation and fragmentation of welfare services, creating a need for integration between different professions, organizations and sectors [1,2]. This is not least the case in transitional care of the elderly.

Elderly persons often have complex needs, requiring services from different providers, particularly within health care and social services. This means that professionals from a number of organizations and sectors are involved in the care of the elderly, leading to a risk of duplications, inconsistencies or discontinuities in the services provided. Transitions of older patients between different settings have also been associated with failures in care plans and treatment, and strategies to improve transitional care have been lacking [3]. As a result, the patients’ needs for medical care and support may not be satisfied or even attended to. They may also receive excessive treatment or fall between the stools of different providers. Moreover, there may be quality and patient safety problems as well as inefficient use of limited resources [4].

In order to counteract such a development, there is a need for collaboration between professionals and organizations working with care or services for the elderly. There are different strategies to achieve or improve collaboration and quality of transitional care. Systematic reviews indicate a range of models of involvement of hospital and municipal health services in the discharge process of elderly patients [5]. One overall strategy has been to initiate organizational changes, for example by merging organizations or creating new units in order to reduce bottlenecks or discontinuities between different activities or services. In the care of the elderly, observation units and units for intermediate care and early discharge from hospitals have also been developed [6,7,8]. Such units can for example be organized as community hospitals or nurse-led wards.

In transitional care, the quality is strongly related to the transfer itself. Studies indicate that inappropriate medical prescriptions have been prevalent among older people acutely admitted to hospital, and that the prevalence was not reduced during a stay at an intermediate unit especially designed for the care of these patients [9]. In order to improve transitions of elderly patients and be integrated parts of coordinated clinical pathways, considerable efforts in establishing procedures and routines are required by the organizations involved [10]. For example, standardized patient transfer forms and medication lists have improved the communication in transfers of frail older patients between nursing homes and hospitals [3].

In addition to organizational change and improvement of specific procedures, there are also different models for collaboration between existing organizations. There are structural models like case or care management, different forms of partnerships, co-location of professionals from different organizations, or financial coordination. There are also process-oriented models for information exchange, more or less systematic interprofessional or interorganizational meetings, or different forms of teamwork. These models are used not only for collaboration in elderly care, but also for example in psychiatric care and vocational rehabilitation. They can also be combined in different ways [11,12]. In the care of the elderly, collaboration between health care settings has been associated with failure in communication and the transfer of essential information, and a growing evidence indicates a correlation of patient handovers with medical errors, adverse events and fragmented care [3,13,14]. In this context, different institutional values can hamper the collaboration [10]. Therefore, cultural issues are also imbedded in models of interorganizational collaboration.

Transitional care has become one of the most pressing topics in the global efforts to improve quality of health services and patient safety. This has been defined as a set of actions ensuring the coordination and continuity of healthcare as patients transfer among different locations and different levels of care within the same location [13,15]. In these settings, good patient handovers require considerable efforts by the individuals and organizations involved to make the patient transitions to integral and vital components of quality and safety [13,16]. However, to promote high quality and patient-centred care, and to prevent adverse events in transfers, a broader notion of collaborative practice is required. This includes the involvement of patients, family members and other informal caregivers [14,17]. In particular, intermediate care should involve patients and their carers in collaborative decision-making concerning their care and the place of care [18]. The focus of this article is on interprofessional and interorganizational collaboration.

### The Norwegian context

In Norway there has been a need for integration primarily in the field of health care, but also in other welfare services. In 2006 there was a merger of the employment service and social insurance combined with an increased collaboration between these agencies and the municipal social services within the framework of the Norwegian Labour and Welfare Administration. In health care, there is an ongoing reform to improve collaboration between the municipal health and social services and particularly the specialized health services of the hospitals, which are organized in the form of health enterprises.

The planning of this reform started in 2005, when a parliamentary commission presented proposals for coordinated and seamless health services [19]. As a result, *The Coordination Reform* for the health services was passed by the parliament [20]. The reform has been gradually implemented from 2012, and involves a duty for the municipalities and the health enterprises to collaborate in the care of elderly patients. It also involves financial incentives for the municipalities to establish appropriate care and services and to promote health for elderly patients who are ready to be discharged from hospitals. The municipalities have the main responsibility for these patients from the first day after their hospital treatment is finished.

The coordination reform was also a starting point for introducing mandatory service agreements between collaborating municipalities and health enterprises, which are required to have legally binding agreements since 2012. Studies of first-generation agreements indicate that mutual sharing of knowledge can be significant for improving the interorganizational collaboration, in response to patients’ needs for coordinated care [21]. From 2016, the municipalities have to provide services for elderly patients who need emergency help or observation.

Thus, the Norwegian coordination reform involves structural changes, financial incentives and legal obligations to improve interorganizational collaboration between municipalities and health enterprises. The question is what their duty to collaborate really means. Experiences from other fields of collaboration show that it is difficult to force organizations to collaborate if not all of them can see some “collaborative advantage” [22]. In addition, these researchers argue that trust building in collaborative relations can be problematic. Therefore, practitioners need to engage in a continuous process of nurturing the collaborative processes, to build trust in situations where this is possible and to cope with situations where trust is lacking [23].

Experiences also indicate that collaboration can be established if all the professionals involved have a strong commitment to the patients served. Such a commitment cannot be decided from above, but has to grow from below over a longer period of time [24]. Interprofessional collaboration is a cornerstone in the development of interorganizational and intersectorial collaboration. Such a cumulative perspective can also be traced in the Norwegian reform.

### The collaboration project

Before the implementation of the coordination reform, a three-year trial with a post-discharge programme in an intermediate ward for transitional care was initiated in a Norwegian community. The programme was established in April 2009 as a collaboration project between a municipality and a hospital that was part of a health enterprise. The intermediate ward provided care for elderly patients in transition from the hospital to the municipal health services, and the objective was that the patients after their stay would be able to return to their homes. The ward had eight beds divided into four rooms with two beds in each. There were 13 employees, most of them nurses and auxiliary nurses, but also a physician and a physiotherapist who were working part-time. The ward was located in a municipal nursing home and the personnel was employed by the municipality.

The collaboration project was based on an agreement between the municipality and the health enterprise. According to this agreement, the intermediate ward would receive patients who had been diagnosed and given most of their treatment at the hospital, so they could be sent home after two or maximum three weeks of transitional care at the ward. There were restrictions, however, that these patients should be citizens of the municipality, 60 years of age or older, and not be suffering from dementia. There was a project leader who was also the nursing manager of the intermediate ward. In addition, there was a project group and a steering group with managers from the municipal administration and the health enterprise. The collaboration between the two organizations was taking place in these groups and also in the daily contacts between the personnel at the intermediate ward and at the hospital departments involved.

Experiences from similar projects in Norway indicate that establishing intermediate wards can be challenging. Particularly, to see their own organizations as a part of a greater whole can be hard for the collaborating partners [10]. Other Norwegian studies show that such wards can reduce the coordination challenges during discharge of elderly patients and relieve the pressure on hospitals and municipal nursing homes, while the impact on the utilization of primary health care was minor [25,26]. Such wards can reduce the total costs of care. Moreover, elderly patients seem to be more positive towards transitional care and have less need for home care and other forms of municipal support after a stay at an intermediate ward [25,27,28,29]. Earlier experiences from other countries point in the same direction [e.g. 30]. Recent reviews emphasize the significance of intermediate units in elderly care, but views of patients and carers represent a gap in the research literature [8].

Against this background, the aim of this study was to describe and analyse the collaboration between the municipality and the health enterprise on different organizational levels. The focus was on the process of collaboration and its possible effects on the quality of care and the economic efficiency of the collaboration for the organizations involved. The quality assessment included aspects of patients’ satisfaction with their stay at the hospital and the intermediate ward, which they were discharged to, and their subsequent needs for home care or support. The economic assessment included the length of stay and the average costs of patients at the ward and at the hospital, but also the costs for home care and support from the municipality. Analysis of collaboration, quality and efficiency also involved issues of trust and integrity.

## Methods

A mixed method design is comprised of a qualitative or quantitative core component that directs the theoretical drive, with one or more qualitative or quantitative supplementary components [31]. Such a design is preferable when mixed methods are likely to provide findings and outcomes in relation to specific research questions [32]. In this study, qualitative methods were the drivers. The collection of qualitative and quantitative data started at the same time. This can be regarded as a convergent parallel form of mixed methods [33].

The process of collaboration between the municipality and the health enterprise was studied through a collection and analysis of written documents like project plans, agreements, annual reports, notes and minutes from meetings, statistics and information materials. In addition, a number of persons from the municipality and the health enterprise were interviewed. Some of these were selected strategically, others successively through “snowball sampling” [34].

The interviews were made in 2011, during different periods of the year. There were 31 interviews with 28 participants, who were in different ways involved in the collaboration project. Among the participants were nine from different clinical departments at the hospital, two from the top administration, one from the board of the health enterprise, six from the intermediate ward, six from the municipal administration, three politicians from the municipality, and one participant from the local university college who had been involved in the establishment of the ward. Three of the participants were interviewed twice.

All the interviews were made by two of the authors and conducted as informal conversations with the interviewees at their workplace. They were following an interview guide with four thematic areas: the collaboration between the hospital departments and the intermediate ward, the collaboration between the health enterprise and the municipality, the barriers and the facilitators in the collaboration. Each interview lasted between one and two hours and was documented in parallel notes by both interviewers. These notes were transcribed and a thematic content analysis was performed in a process where different impressions and interpretations of the two authors were compared [35]. The common themes were also discussed with the other authors in order to increase the credibility of the findings. Illustrative quotations were extracted and agreed between the authors. In this way, the interview results were also validated in accordance with qualitative methodology [36].

The effects on the quality of care were assessed in a retrospective and a prospective study of patients at the intermediate ward and the hospital. The retrospective study was conducted by means of a postal questionnaire sent to all the patients who had stayed at the intermediate ward in 2010. There were questions about their satisfaction with different aspects of the care received, and also questions about where they had been discharged to after their stay at the ward and their needs for municipal care or services when they returned to their homes. The questionnaire was developed and validated by the Norwegian Knowledge Centre for the Health Services, but some adjustments were necessary.

The prospective study was conducted mainly during 2011 and 2012 following a quasi-experimental research design, where a study group of patients who had been transferred from the hospital to the intermediate ward was compared with a control group of similar patients at the hospital. Because of the small number of patients at the ward it was not possible to make a random selection of patients to the two groups. Instead, the control group was composed of patients who fulfilled all the criteria for admission to the ward except that they were not citizens of the municipality. The patients in the two groups were compared using a similar questionnaire as in the retrospective study.

The results from the retrospective study (n = 62) and some of the results from the prospective study were analysed using descriptive statistics [37]. For the prospective study, the comparisons between the study group (n = 58) and the control group (n = 30) were made mainly by using independent samples t-tests, in some cases supplemented by independent samples median tests. The question of where the patients were discharged to was analysed using cross tabulation and chi-square test.

The question of whether the patients in the study group were more satisfied with their stay at the intermediate ward than at the hospital was analysed using a paired samples t-test. Because some respondents did not answer all questions, the sample sizes vary slightly in the analysis of the different questions. Post hoc power analysis was conducted, with significance level set to the conventional p = 0.05.

The Regional Committee for Medical and Health Research Ethics approved both studies in December 2010. They were part of a larger research project on quality in the care of elderly patients, which will be reported in more detail elsewhere.

The economic effects were studied mainly through an analysis of the length of stay and the average costs per patient and day at the intermediate ward compared to the average costs per patient and day of the clinical departments at the hospital. This analysis was based on financial reports and official statistics from the municipality and the health enterprise. However, because of the small number of patients involved, it proved difficult on the basis of this information to calculate any direct savings for the health enterprise by transferring patients from the hospital to the intermediate ward.

There were great differences in average costs between different hospital departments and also between different phases of treatment. Therefore, only departments with similar patients to the intermediate ward should be considered and only the last phases of treatment. There are also alternative costs in terms of lost revenues for new patients due to bed occupancy at the hospital that should be considered. It was not possible, however, to extract any information on alternative costs and average costs during the last phases of treatment from the financial systems. Instead, the potential savings of the health enterprise were estimated by comparing the average costs of patients at the ward with the average costs of similar patients at the hospital departments.

In the same way, it proved difficult to calculate any direct savings for the municipality on the basis of information from the existing financial systems, because of the small number of persons involved. Therefore, a simple questionnaire was constructed by the researchers and distributed by the municipal administration to all the clinical managers within the municipal home care. There were questions concerning the needs for care and support of elderly persons who had been staying at the intermediate ward compared to persons who had been sent home directly from the hospital. Based on this questionnaire, it was possible to discuss the potential savings of the collaboration project for the municipality.

## Results

### The process of interorganizational collaboration

The initiative to the collaboration project came from the top managers of the health enterprise, who heard about the positive experiences of intermediate wards for transitional care in other parts of the country. The health enterprise financed 2/3 of the project, while the remaining 1/3 was financed by the municipality. In spite of the different financial contributions to the project, the collaboration between the health enterprise and the municipality worked well on the top level of the two organizations.

There was a good climate of collaboration in the project group and the steering group, where different managers represented both parties. The politicians were also very supportive of the collaboration project. All of them saw a great value in a common project like this. According to one of the managers, *“it is valuable to test the collaboration between the municipality and the health enterprise, as it will anyway become a duty in connection with the coming coordination reform”.* An important facilitating factor was that none of the managers saw it as a loss of prestige if the project should not turn out to be successful. *“We will in any case learn from the experiences”.*

Unlike the management relations, the contact between the personnel at the intermediate ward and the hospital departments was more problematic. This was the case particularly in the contacts between the ward and the departments of general surgery and internal medicine. Many of the interview persons from these departments felt that this project was decided from above and forced upon them. In their opinion, the intermediate ward should be closed down immediately. As one of them pointed out, *“the ward is expensive and a waste of resources that could be used better at the hospital”.* Moreover, they did not trust the personnel at the ward. *“They do not have the competence required to take care of our patients”.*

The personnel at the hospital departments saw a number of barriers to collaboration. The strict criteria for admission to the intermediate ward made it difficult to find patients at the hospital who could be transferred there. Even when such patients were identified, the ward was sometimes unable to receive them. This could be due to a lack of personnel at the ward, or because it was impossible to place male and female patients in the same room. When the ward refused to receive patients who fulfilled all the admission criteria, the personnel at the hospital departments felt themselves cheated. It was also difficult for them to reach the personnel at the ward and discuss these matters because of limited telephone hours. The result was increasing annoyance and a hesitation to send patients to the ward.

The personnel at the intermediate ward had a positive view of the collaboration project, but they felt that there was a strong resistance from some of the departments at the hospital. They experienced that the personnel at these departments were badly informed about the project and the agreements between the municipality and the health enterprise. This was an important barrier to collaboration. They also felt that the personnel at the hospital departments were cheating them. According to one of the nurses at the intermediate ward, *“they want to get rid of certain patients and when we are not accepting them, they are punishing us by not sending any patients at all”.*

Thus, while both the managers of the municipality and the personnel at the intermediate ward had a positive view of the collaboration project, there were different opinions among the managers of the health enterprise and the managers and personnel at some of the hospital departments. The managers at these departments were not able to convince their personnel of the needs and the usefulness of an intermediate ward, which might have facilitated the collaboration with the personnel at the ward. Additionally, they were not able to order the hospital personnel to collaborate. There was not a great deal of interest in the project, particularly among the physicians at the hospital. According to one of the managers in the project group, *“there were information meetings where no one came”*.

The contacts and communication between the hospital departments and the intermediate ward were problematic from the start of the project. There were signs, however, that they were overcoming some of the barriers and that their collaboration and relations of trust were slowly improving. There were more contacts between the part-time physician at the intermediate ward and the physicians at the hospital. The contacts between the personnel at the ward and the hospital were also improving. There were mutual adjustments of the routines for transferring patients from the hospital, and the telephone hours of the ward were extended. In addition, there was an increased understanding of the advantages of the intermediate ward. As one of the nurses at the hospital admitted, *“it is probably more expensive and worse for the patients to stay at the hospital”.*

### Effects on the quality of care

According to the results of the retrospective study, 95 % of the patients who returned the questionnaire (n = 62) were satisfied or very much satisfied with their stay at the intermediate ward. They appreciated particularly the professional competence of the personnel, their concern for the patients and the quality of the food.

The patients in the study group, who were treated both at the hospital and the intermediate ward (n = 52), were less satisfied with their treatment and care at the hospital than the patients in the control group (n = 28), and more satisfied with their treatment and care at the intermediate ward than at the hospital, although the differences were not statistically significant (p = 0,595 and p = 0,063). However, the group of patients who were treated both at the hospital and the intermediate ward were slightly less satisfied with their total treatment and care (ward and hospital) than the patients in the control group, although the difference was not statistically significant (p = 0,287).

The patients’ satisfaction with different aspects of treatment and care was also analysed, and the results are summarized in Table 1.

**Table 1:** Prospective study: satisfaction with aspects of treatment and care.

Aspects of treatment and care	Study group		Control group
	Percentage of patients who were satisfied or very much satisfied:		Percentage of patients who were satisfied or very much satisfied:
	Hospital	Intermediate ward	Hospital

Professional competence	79 %	86 %	93 %
Concern for the patients	73 %	90 %	100 %
Information on treatment	58 %	64 %	73 %
Involvement of patients	29 %	53 %	47 %
Organisation of care	55 %	81 %	77 %
Medical equipment	74 %	79 %	60 %
Peaceful environment	60 %	79 %	70 %
Personnel-patient time	58 %	84 %	87 %
Food and meals	58 %	76 %	70 %
Reception of relatives	71 %	82 %	83 %
Information on discharge	68 %	80 %	77 %

**Figure 1:**
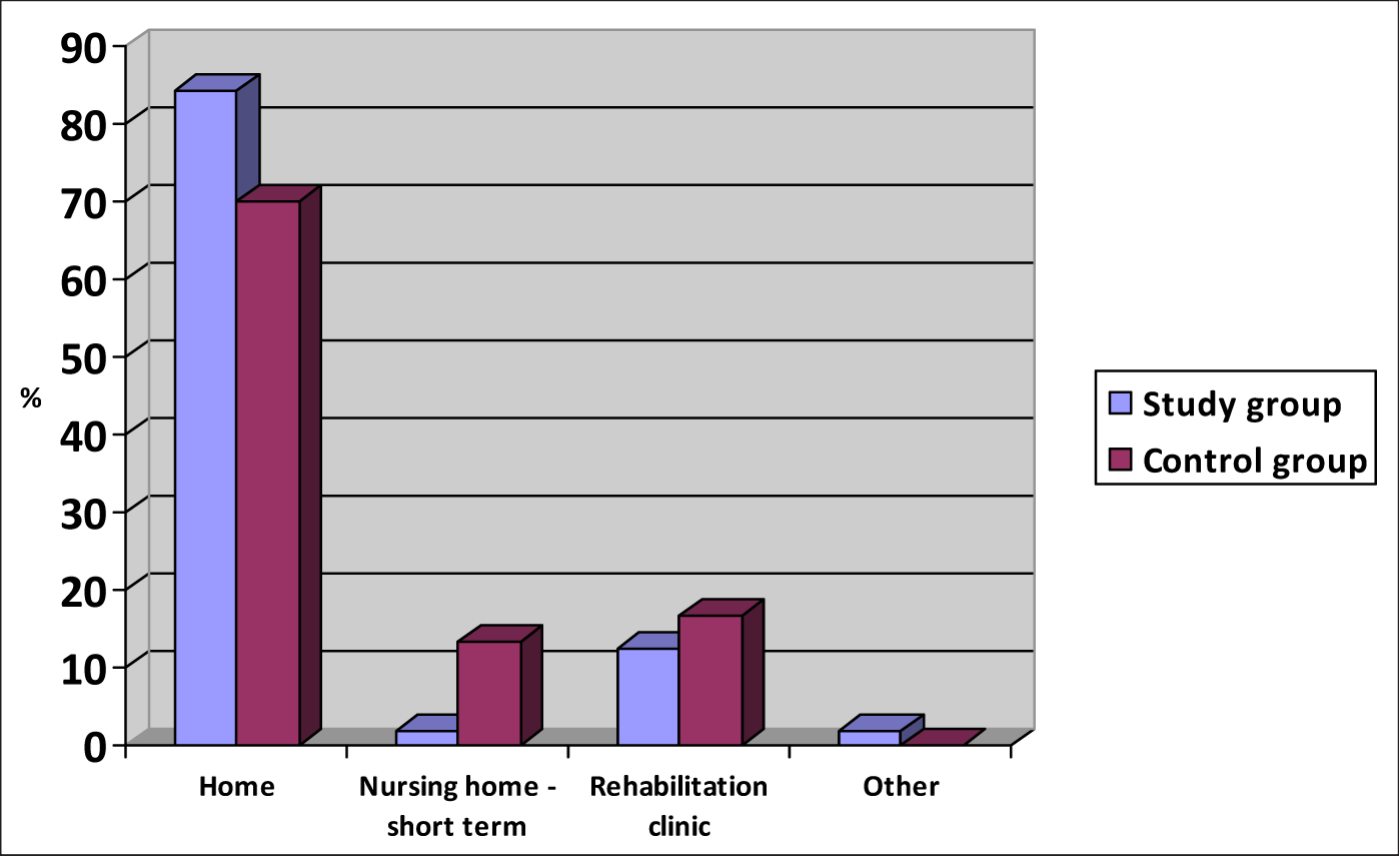
Where the patients were discharged to.

Regarding readmission and discharge, only 3% of the patients in the retrospective study were readmitted to the hospital after their stay at the intermediate ward. In the prospective study, there was a low rate of hospital readmission among both the study group and the control group. However, results from the prospective study show that the patients who were transferred to the intermediate ward (n = 57) could return to their own homes more often than the patients in the control group (n = 30), although the difference between the groups was not statistically significant (p = 0,12). A larger percentage of the patients from the hospital were instead sent to short term stays at nursing homes, or rehabilitation clinics. These results are summarized in Figure 1.

According to the retrospective study, 62.5% of the patients who were transferred to the intermediate ward had some needs for municipal care or services when they returned to their homes. Results from the prospective study show that the patients who were discharged from the intermediate ward (n = 50) experienced a greater need for home care and services than the patients in the control group (n = 23), and this difference is statistically significant (p = 0,022). For the given effect size, statistical power is 0,640. The results also show, however, that the control group received help and care from a larger number of providers once they were home than the study group. This difference is also statistically significant (p = 0,008). For the given effect size, the statistical power is 0,766.

### Economic effects

During the period studied, the patients at the intermediate ward stayed for an average length of 12.2 days before they were sent home. This length of stay is within the two or maximum three weeks that were expected for transitional care at the ward. However, there was great differences in bed occupancy during the period studied. On the average, the bed occupancy was only 68% during 2009 and 2010, but it increased to 82% in 2011. Even so, the beds at the intermediate ward were much less occupied than the beds at the hospital. During the same period, the departments of general surgery, orthopaedic surgery and internal medicine had a constant bed occupancy rate of 100%, which means that there was a great pressure on the hospital beds and also a need to relieve this pressure.

Table 2 shows the average costs per patient and day at the intermediate ward compared with the three hospital departments that were transferring most of the patients. The costs included salaries, food and medication, premises, equipment, electricity and municipal charges. Even with a bed occupancy rate of 70%, the average costs of the intermediate ward were lower than the average costs of the hospital departments, except the department of general surgery. With a similar rate of bed occupancy as the departments of the hospital, the average costs of the intermediate ward would have been much lower.

**Table 2:** Average costs (NOK) of patients at the intermediate ward and the hospital departments.

Units	Bed occupancy	2009	2010	2011

Department of general surgery	100 %	4.044	4.240	4.157
Department of orthopaedic surgery	100 %	4.501	4.742	4.841
Department of internal medicine	100 %	5.360	5.921	6.137
Intermediate ward	70 %	4.088	4.282	3.809
Intermediate ward	100 %	2.862	2.998	2.667

This means that the costs of the health enterprise could be reduced by transferring patients from the hospital to the intermediate ward, although such savings were not possible to extract from the financial information systems. The savings would be even greater with higher rates of bed occupancy at the ward. This could also relieve the pressure on the hospital beds, or make it possible for the hospital to admit new patients, which would generate more revenues for the health enterprise.

The intermediate ward may also have brought some savings for the municipality. The patients from the intermediate ward were more often discharged to their own home, which in most cases is less costly for the municipality than a discharge to a municipal institution. At the same time, they seemed to require more help and support in their homes than the patients in the control group, although the results are inconclusive. However, a questionnaire answered by all the clinical managers of the municipal home care indicated that there were many different needs for care and services, depending on the individuals concerned. A common view was that patients from the hospital were more demanding than patients from the intermediate ward. In addition, the home care had better collaboration with the ward than with the hospital departments. Although it is difficult to draw a firm conclusion from the available data, it seems that the intermediate ward may have brought some financial gains to the municipality.

## Discussion

This study has shown that the collaboration between the municipality and the health enterprise was working well on the top management level of the two organizations. It was more problematic on the operative level between the personnel at the intermediate ward and the clinical departments at the hospital. The collaboration between the managers of the municipality and the health enterprise was characterised by mutual interest in and support of the project, while the contact between the personnel at the intermediate ward and the hospital departments was characterised mainly by conflicts and lack of trust. The managers could see some collaborative advantage in the project and so could also the personnel at the intermediate ward, while the personnel at the hospital felt that the project was forced upon them and that it was a waste of time and resources.

There were clear signs, however, that the collaboration problems were reduced. The trust was increasing during the time of the project through improved communication, adjustments of transfer routines and an increased understanding of the mutual advantages and challenges of collaboration. In addition, the bed occupancy rate of the intermediate ward was increasing, probably as a result of better information and improved collaboration between the personnel at the intermediate ward and the hospital departments. This development is not surprising. Reviews of international research in the field show that it is usually a long process and takes a lot of time and energy to establish and maintain collaboration [1,12]. In economic terms, collaboration “costs before it pays” [38]. Effects of interorganizational collaboration should therefore not be judged merely in terms of short-term gains, but also in a longer-term perspective. This also points forward to the concept of organizational health, where dimensions of integration and collaboration are embedded [39].

Researchers dealing with care of the elderly point out that there are often problems with continuity and quality of care as well as economic efficiency [3,4]. Many of these problems can be traced to a lack of trust and collaboration between the different professions and organizations involved [23]. Therefore, intermediate wards or observation units have been suggested as links between health care and social services of the elderly [6,7,40]. In addition, different models have been developed for collaboration between health care and social services [e.g. 41,42,43]. In the project studied, the intermediate ward was meant to facilitate early discharge of elderly patients from the hospital. At the same time, a model for collaboration was developed, with objectives related to the quality of care as well as economic efficiency.

The assessment of quality of care showed that the patients on the whole were satisfied with their stay at the intermediate ward. They were also slightly less satisfied with their stay at the hospital than the patients who completed their treatment and care there. Earlier studies of patient satisfaction have shown that such positive results are not unusual [44,45,46]. Thus, the collaboration project may have improved the client-perceived quality and so the user involvement, which has been characterized as a specific feature of an elderly care gaining its own identity [43].

In addition, the patients from the ward were more often discharged to their homes after their stay, and were perceived by the home care managers to have a higher level of functioning than the patients from the hospital. However, they seemed to have significant more need for municipal care and support compared with patients who were discharged directly from the hospital, but received help and care from a lower number of providers once they were home.

The economic assessment showed that the intermediate ward had lower average costs per patient and day than the departments at the hospital. Even if the ward in certain periods had not been able to fill their beds, meaning that the costs of the ward were divided between fewer patients, the average costs of the ward were still lower than the average costs of the hospital departments.

It was difficult to show any direct savings for the health enterprise and the municipality. Even so, there seemed to be positive economic effects of transferring patients to the intermediate ward before they were sent home. This may have reduced the pressure on the hospital beds, even if it is was not possible to show in this study. It may also have reduced the pressure on temporary care in nursing homes, as the patients from the ward were discharged to their own home more often than the patients from the hospital. At the same time, however, they needed more care and support than the patients from the hospital after returning home. Thus, it is difficult to show any direct savings for the municipality.

Other studies have indicated that patients had a higher level of functioning after a stay at intermediate wards than the patients who were discharged directly from hospitals [47]. In this study, the patients from the ward were perceived to have a higher level of functioning than the patients from the hospital. On this point, however, the results were inconclusive.

In addition to effects on quality and efficiency, the data indicated both positive and negative collaborating relations. As care and transfer of elderly patients may be characterized by a high degree of differentiation and fragmentation, a high level of integration is required. There were positive contacts and a state of integration between managers and units. For example, in the project group and the steering group, representatives from both organizations had a good climate of collaboration. However, the interface between hospital departments and the intermediate ward was characterized by antagonistic contacts and conflicts, which is a state of disintegration. As suggested, the personnel at the ward felt themselves cheated by colleagues at the hospital departments. They even suggested that their colleagues wanted to punish them by not sending any patients at all. Seemingly, a bad climate may be destructive and compromise the interprofessional and interorganizational collaboration. However, a state of disintegration can also be necessary to manage conflicts and contribute to negotiations and integration.

Findings of good and bad relations are not surprising, as facilitators and barriers of interorganizational collaboration are typically connected with factors of communication, commitment and trust [12]. For example, clinical personnel at the hospital felt that the collaboration project was forced upon them and that it was a waste of time, and considered their colleagues at the intermediate ward not to be qualified to take care of the transferred patients. Such reactions might be a form of resistance to change. However, incongruent clinical considerations and the feelings of being disintegrated in the collaboration project could also reflect an unwillingness to perform a work that was not in accordance with personal values. If so, resistance could be a way of maintaining integrity.

Over time, feelings of being alienated to interorganizational processes and forced to do what you do not want to do may induce integrity pressure with potentially negative effects on work health and wellbeing [48]. Integrity pressure may, however, also have implications beyond individual health. In this context, the concept of organizational health can be valid [39]. The connection between interorganizational collaboration and organizational health may even point forward to a concept of interorganizational health.

As mentioned before, research indicates that it takes a lot of time and energy to establish and maintain collaboration [24]. A continuation of the project should therefore have been combined with activities to improve trust, communication and mutual understanding between the hospital departments and the intermediate ward. The study indicated that the criteria for admission of patients to the ward were not clear and should be revised. There should also have been a development of collaboration between neighbouring municipalities, which would probably have increased the recruitment of patients to the intermediate ward and at the same time also decreased the average costs for patients at the ward. Such joint efforts can facilitate interorganizational collaboration and even promote interorganizational health.

## Conclusions

This article reports a study of a post-discharge programme for elderly patients in Norway. The programme took place in an intermediate ward for transitional care, which was based on collaboration between a municipality and a hospital belonging to a health enterprise. The patients on the whole were satisfied with their stay at the ward and most of them could be sent home after two weeks of transitional care.

The intermediate ward had lower average costs than the clinical departments at the hospital. This means that there could be positive economic effects for the health enterprise by transferring patients to the ward. There were probably positive economic effects also for the municipality, as the patients from the ward were discharged to their homes rather than to short term stays at municipal institutions, which in most cases is less costly for the municipality. Although they seemed to have a greater need for care and support in their homes than the patients who were discharged from the hospital, the patients from the ward were perceived by the municipal team managers to have a higher level of functioning.

There were a number of barriers to collaboration on the operative level, related both to formal obstacles, lack of trust and negative attitudes to collaboration among the health personnel. There were, however, signs of mutual adjustments and improved collaboration between the two groups of personnel, which indicate that the development of collaboration has to grow from below over a longer period of time. Positive and negative collaborating relations impacted values of quality and efficiency, and also made current issues of trust and integrity. Thus, interorganizational collaboration can also be related to interorganizational health.

In spite of increasingly and mainly positive results in an atmosphere of improving collaboration, the board of the health enterprise decided to close down the intermediate ward in 2012. This decision was based mainly on short-term intraorganizational and economic considerations. Although the intermediate ward was closed down, the findings of the study suggest that a continuation of this interorganizational collaboration might have had positive long-term effects on the quality of elderly care and also on the economic situation of both the health enterprise and the municipality.
